# Bilateral germ-cell tumours: 22-year experience at the Institut Gustave Roussy

**DOI:** 10.1038/sj.bjc.6601464

**Published:** 2004-01-06

**Authors:** Ch Theodore, M J Terrier-Lacombe, A Laplanche, G Benoit, K Fizazi, O Stamerra, P Wibault

**Affiliations:** 1 Department of Medicine, Institut Gustave Roussy, 39, rue Camille Desmoulins, 94800 Villejuif, France; 2 Hopital de Bicêtre, Le Kremlin Bicêtre, France

**Keywords:** incidence, bilateral testicular germ-cell tumours, seminoma, nonseminomatous germ-cell tumour

## Abstract

The aim of this study was to describe the incidence, clinical and histological characteristics, treatment and long-term follow-up of bilateral germ-cell tumours (BGCT) of the testis in order to determine in what respects they differ significantly from unilateral germ-cell tumours. In all, 31 patients with BGCT had metachronous tumours and 14 had synchronous tumours. Among the metachronous tumours, 61% occurred more than 5 years after the first tumour. The overall incidence of BGCT in patients with testicular germ-cell tumours (TGCT) was 1.9%. The incidence was 3.2% in patients presenting with a seminoma and 1.4 % in patients presenting with a nonseminomatous germ-cell tumour (NSGCT). Patients under 30 years of age at the time of the initial diagnosis had a higher incidence of bilateral tumours compared with older men. The outcome of BGCT was excellent. A high association was found between BGCT, sterility and suspected genetic risk factors for TGCT. These results argue against a systematic contralateral biopsy at diagnosis of first TGCT in all patients, but emphasise the importance of patient education and of the need to better identify patients at risk for a second TGCT. Therapeutic indications for synchronous BGCT, including conservative treatment, need to be better defined.

Testicular germ-cell tumour (TGCT) is a rare disease with an estimated incidence of five out of 100 000. An increase of more than 50% has been demonstrated in all Western countries during the last decades (Grenlee *et al*, 2001). Several risk factors for developing a TGCT have been identified, uppermost among which is a previous TGCT (Osterlind *et al*, 1991). The incidence of bilateral testicular germ-cell tumour (BGCT) ranges between 1 and 5% in previously published large series (Bokemeyer *et al*, 1993; Dieckmann *et al*, 1993, 1999, 2002; Heidenreich *et al*, 1995, 1997, 2000; Gerl *et al*, 1997; Tekin *et al*, 2000; Geczi *et al*, 2001; Ondrus *et al*, 2001; Che *et al*, 2002; Ohyama *et al*, 2002). The charts of 2383 consecutive patients treated at the Institut Gustave Roussy over a 22-year period were analysed to estimate the incidence of BGCT, to identify potential risk factors and to evaluate the long-term survival of patients with BGCT.

## PATIENTS AND METHODS

Between 1979 and 2002, 2383 patients with TGCT were treated in the Adult Cancer Department at the Institut Gustave Roussy (IGR). The list of patients was obtained from a computerised database. Among these 2383 patients, 45 had a BGCT. The medical records of these 45 patients were reviewed to collect the following information: previous personal and family medical history, clinical characteristics at presentation, histopathology, treatment, interval between the development of the two tumours and follow-up.

All available pathology slides were reviewed. Most patients had their orchidectomy at other institutions. Haematoxylin- and eosin-stained slides of both testicular tumours were available for 36 patients, and slides of only one tumour were available for three patients. A pathology report was available in all cases.

All tumours were staged according to the TNM and the ICCCG staging systems, which take into account the extent of the primary tumour, retroperitoneal lymph nodes, distant metastases and serum markers.

All patients were divided into two groups based on the histological subtype of their testicular tumour: the seminoma group comprised only patients with pure seminoma, and the NSGCT group comprised patients with any mixture of germ-cell tumour as long as one of the components was nonseminomatous and any pure form of germ-cell tumour other than seminoma. Patients were then divided into three groups according to their age: 15–30, 30–45 and over 45 years. Patients' treatment was based on the histologic type and clinical stage.

All patients treated before 2001 had a bilateral orchidectomy. Most patients with seminoma confined to the testis or with small involved retroperitoneal lymph nodes had radiation therapy after the orchidectomy, whereas most patients with either advanced seminoma (with large retroperitoneal masses and/or distant metastases) and most patients with NSGCT had chemotherapy after orchidectomy. Whereas patients with NSGCT treated before 1985 had a systematic retroperitoneal lymph-node dissection (RPND), this surgical procedure was restricted to patients with residual masses after chemotherapy after 1985. Data were analysed using the χ^2^ test ; *P*-values of less than 0.05 were considered to be statistically significant.

## RESULTS

In all, 31 of the 45 patients had metachronous tumours and 14 patients had synchronous tumours. The median age was 26 (range 16–58). Nine patients had a history of cryptorchidism that was bilateral in five, three had a family history of TGCT, one had a family history of bilateral testicular cryptorchidism, seven had atypical nevi, one had Down's syndrome, one had piebaldism and one had a subsequent anal squamous cell cancer. In total, 10 were explored for infertility before the diagnosis of the germ-cell tumour, five of whom had a metachronous BGCT and five a synchronous BGCT.

### Pathological features of BGCT

Seminoma was the predominant histology: among the 90 tumours, 54 were seminomas in 36 patients and 36 were NSGCT in 22 patients. Histology was concordant in 27 patients and discordant in 18 patients.

In the 14 patients with synchronous tumours, histology was concordant in 10 cases (nine with seminomas and one with NSGCT) and discordant in four patients.

In the 31 men with metachronous tumours, histology was concordant in 13 cases and discordant in 18: the tumour was a seminoma in 32 testicles and NSGCT in 30 testicles. In total, 14 patients presented with seminoma, nine of whom developed a second seminoma, whereas five had an NSGCT as their second tumour. In all, 17 patients presented with NSGCT, eight of whom had a second NSGCT, whereas nine developed seminoma as their second tumour. There was no correlation in terms of histologic type between the first and the second tumour in men with metachronous tumours.

### Incidence

Of the 2383 patients who were treated at the Institut Gustave Roussy between 1979 and 2002, 45 patients (1.9%) developed a BGCT of the testis.

The incidence of metachronous BGCT was 1.3% and that of synchronous BGCT 0.6%.

The probability of developing a metachronous TGCT was 1.8% (0.9–2.8%. CI 95%) in patients who presented with seminoma and 1.1% (0.6–1.8%. CI 95%) in patients who presented with NSGCT, and this difference was not statistically significant (*P*=0.24) (Table 1

**Table 1:** Histology-specific incidence of metachronous BGCT

**Histology**	**All patients**	**Patients with BGCT**	**%**
Seminoma	836	14	1.8
NSGCT	1547	17	1.1

). However, the incidence of metachronous TGCT in patients with seminoma was markedly influenced by the patient's age at the time of presentation. The probability of developing a metachronous BGCT was 3.9% (1.8–7.2%. CI 95%) in patients who presented with seminoma under the age of 30 years and 0.8% (0.3–1.9% CI 95%) in patients older than 30 years who presented with seminoma and this difference was statistically significant (*P*⩽0.01). (Table 2

**Table 2:** Age-specific incidence of metachronous BGCT in patients presenting with seminoma

**Age group**	**All patients with seminoma**	**Patients Nb**	**BGCT %**
15–30	232	9	3.9
>30	604	5	1.3
Total	836	14	1.7

). The incidence of metachronous BGCT was also influenced by age in patients with NSGCT with a probability of developing a metachronous TGCT of 1.5% (0.8–2.5% CI 95%) in the ⩽30 years age group and 0.5% (0.1–1.5%. CI 95%) in the ⩾30 age group. However, this difference was not statistically significant (*P*=0.08) (Table 3

**Table 3:** Age-specific incidence of metachronous BGCT in patients with NSGCT

**Age group**	**All patients with NSGCT**	**Patients Nb**	**BGCT %**
30–45	946	14	1.5
>30	601	3	0.5
Total	1547	17	1

).

In the whole population with synchronous and metachronous BGCT, 36 patients had at least one seminoma (out of 836 patients treated for seminoma), 17 of whom were ⩽30 when the seminoma was diagnosed (out of 232 patients treated for seminoma ⩽30). The general incidence of BGCT in the seminoma patient population was 4.3% (3–5.9%. CI 95%) and 7.3% (4.3–11.5%. CI 95%) in patients with seminoma ⩽30.

### Intervals between the first and the second tumour

**Figure 1:**
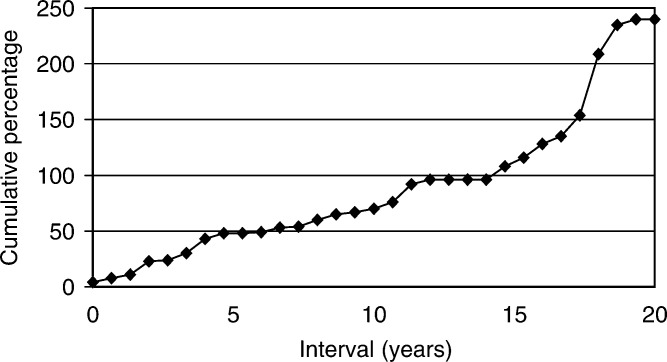
Time intervals between first and second tumours in the 31 patients with metachronous bilateral testicular germ-cell tumours.

In 31 patients with metachronous BGCT, the median interval between the first and the second tumour was 60 months (range 4–240). The second tumour occurred within 5 years in 13 patients (41%) and between 10 and 20 years in seven patients (23%).(Figure 1

). It is noteworthy that nine patients had received Cisplatin-based chemotherapy (VAB 6 in five patients, BEP in two, EP in one and CISCA in one) during treatment of their first TGCT.

The median interval between the first and the second tumour was 70 months (range: 43–154) in these patients.

### Clinical features, treatment and outcome

Among the 31 patients with metachronous BGCT, the first tumour was TNM stage I in 23 patients, stage II in seven patients, one IGCCCG intermediate-risk BGCT and one IGCCCG good-risk stage III BGCT. The treatment was radiotherapy in 14 patients, retroperiteal lymph-node dissection (RPLND) alone in three patients, surveillance after orchiectomy in five patients and chemotherapy, possibly followed by RPLND, in nine patients. The second tumour was stage I in 25 patients, stage II in three patients and stage III in three patients, IGCCCG poor prognosis in one patient. The treatment was radiotherapy in six patients, RPLND alone in two patients, surveillance after orchiectomy in 10 patients and chemotherapy in 13 patients.

Among the 14 synchronous BGCT, there was one stage I and four stage II tumours, two of which were bilateral seminomas. One patient had an IGCCCG intermediate-risk NSGCT. The treatment was chemotherapy in seven patients and radiotherapy in seven patients.

The median follow-up was 58 months after the second tumour or the BGCT (range 10–218). There was no substantial difference in the TNM stage distribution between patients with seminoma and patients with NSGCT. All 10 patients diagnosed with BGCT after exploration of infertility had limited stage disease (nine patients with stage I and one patient with stage II seminoma).

The overall prognosis of patients with BGCT was excellent as in previous series in the literature (Coogan, Albers). One of the 45 patients with metachronous BGCT presented with poor prognosis disease according to the IGCCCG staging system and ultimately died of refractory metastatic disease. None of the remaining patients had any evidence of disease at the most recent follow-up. Five patients underwent conservative surgery: conservative surgery was restricted to one testis in four patients but performed on both testicles in one patient. One patient had metachronous discordant BGCT with pure mature teratoma in the first tumour at 16 and mixed TGCT with mature teratoma and seminoma, 67 months later in the contralateral testis. The four other patients had bilateral synchronous BGCT, which was bilateral seminoma in three and discordant NSGCT and seminoma in the fourth patient. Adjuvant treatment was chemotherapy with two cycles of BEP in the two patients with NSGCT, chemotherapy with Carboplatin in one patient with seminoma and radiotherapy at a dose of 20 Gy to the ipsilateral lymph nodes and the remaining testicular tissue in two patients including the one patient who had bilateral conservative surgery. All five patients have had regular follow-up consultations with a three-monthly ultrasound and tumour marker determination, and have no evidence of progression in the testis at a median follow-up of 18 months. None of them require substitutive hormone therapy.

## DISCUSSION

It is well known that having a TGCT is the major risk factor for developing a second TGCT in the contralateral testis with the second tumour occurring in 1–5% of patients (Bokemeyer *et al*, 1993; Dieckmann *et al*, 1993, 1999, 2002; Heidenreich *et al*, 1995, 1997, 2000; Gerl *et al*, 1997; Tekin *et al*, 2000; Geczi *et al*, 2001; Ondrus *et al*, 2001; Che *et al*, 2002; Ohyama *et al*, 2002). The first published series by Gilbert and Hamilton (1942) included 1466 consecutive patients and found an incidence of 1.6%. A review of the literature spanning a period of 20 years, published by Aristibal in 1986 found an incidence of 1.56% in 4864 patients. More recently, Bokemeyer found an incidence of 3.5% in 773 patients treated at Hanover University between 1972 and 1985 and Kristiansund reported an incidence of 1.9% in 1300 consecutive patients treated in Norway. Osterlind *et al* (1991); Bokemeyer *et al* (1993); Dieckmann *et al* (1996, 1999, 2002); Gerl *et al* (1997), Geczi *et al* (2001), Che *et al* (2002), respectively, found incidence rates of 2.6% in 2830 patients treated in Denmark between 1960 and 1979, 5% in 181 consecutive patients treated in Berlin, 2% in 787 patients treated between 1979 and 1996 at the University of Munich, 3.5% in 773 patients treated at the University of Hanover, 3% in 2386 consecutive patients treated in Hungary and 1% in 2431 patients treated at the MD Anderson Cancer Centre over a period of 20 years. In all these series, the incidence of synchronous BGCT was lower than that of metachronous BGCT of the testis. Dieckman estimated an incidence of 0.1% in 89 published cases. Che found three in a series of 24 patients with BGCT with an incidence of 0.4%, which is identical to that reported by Bokemeyer. In the large series published by Geczi, the incidence was 0.8%. In our study population of 2383 patients, 14 (0.6%) presented with synchronous BGCT corresponding to an incidence of 0.6% and 31 patients developed metachronous BGCT corresponding to an incidence of 1.3%, in the lower range of the published series (Patel *et al*, 1990, 1993; Park *et al*, 1999; Che *et al*, 2002). The incidence of BGCT may increase in the future due to the development of infertility exploration. Our study supports this premise because 10 of our patients had been explored for infertility. Another reason why the incidence of BGCT may increase could be the long-term survival of patients diagnosed early and who received effective treatment for their first tumour. Our study also supports this premise since seven of the 31 patients with metachronous BGCT developed the second tumour more than 10 years after the first tumour.

The incidence of BGCT has been reported to be different in patients who have seminoma compared with patients who have NSGCT. Osterlind reported a greater incidence in patients who had NSGCT (8.4%) compared with patients who had seminoma (3.6%). The reverse finding was observed by Bokemeyer, who found that 4.8% of patients who had seminoma developed BGCT compared with 1.8% of those with NSGCT and by Che who found that 1.8% of patients with seminoma compared with 0.6% of patients with NSGCT developed BGCT and by Kristianslund. In the present study, there were significantly more BGCT in seminoma patients than in NSGCT patients. However, patients who initially presented with a unilateral seminoma did not have a significantly higher risk of developing a metachronous contralateral TGCT compared to patients who presented with a unilateral NSGCT. The significance of the seminoma histology in the population of patients with BGCT remains unclear.

We also found that the incidence of BGCT in patients with seminoma was influenced by the patient's age at the time of the initial diagnosis. Although seminoma was more common in patients older than 30 years, the incidence of BGCT was much greater in patients in the younger age group. Patients who had an early-onset unilateral seminoma were at greater risk of developing a contralateral TGCT. These results are similar to those published by Che. Dieckman also found that patients with BGCT were younger compared to men with a unilateral tumour, but did not group patients according to histologic subtype. In the present study, the increased incidence of BGCT in younger patients with NSGCT was not statistically significant.

The association of BGCT and suspected risk factors for developing a TGCT has not been systematically reported in most series. Cryptorchidism is the best-known risk factor. Geczi reported the association of BGCT and cryptorchidism in 9.5% of cases, whereas no cases of cryptorchidism were reported in the MD Anderson series (Che *et al*, 2002). Likewise, other conditions reported to be associated with the risk of TGCT such as infertility (Hendenreich), atypical nevi (Raghavan *et al*, 1994; Avril *et al*, 2001) or chromosomal abnormalities such as Down's syndrome (Roberge *et al*, 2001) are not reported in BGCT series. As all these series are retrospective, it is possible that all background data were not recorded in all patient files. The high association with such risk factors demonstrated in this report are suggestive of a genetic predisposition and supports the hypothesis that there may be familial forms of BGCT, like in familial clustering of TGCT (Skotheim, Rapley, Dong *et al*, 2001; Krazggerud *et al*, 2002).

The strategies employed for the management of TGCT were based on the individual patient. (Tables 1 and 2). Treatment decisions for patients with synchronous and metachronous BGCT were based on risk stratification, as defined by the International Germ Cell Cancer Collaborative Group International Germ Cell Cancer Consensus Classification (1997). In patients with metachronous BGCT, only three initially had surveillance for their first NSGCT. One patient relapsed and was treated with chemotherapy. Since nine of the 31 patients received three to six cycles of Cisplatin-based chemotherapy as treatment for their first TGCT, these results show that chemotherapy does not fully protect an individual patient from the development of a second TGCT, unlike that previously speculated (Bokemeyer *et al*, 1993; Park *et al*, 1999). Post-treatment surveillance of these patients consisted in serum tumour marker determinations and imaging studies. The time frame for surveillance was usually defined according to disease presentation. The most common timing of follow-up was evaluation every 3 months for 2 years, every 6 months for the next 3 years and yearly thereafter.

Most of the second tumours were detected during follow-up. However, in some of the patients, the second tumour was detected through self-examination and, in one patient through a metastasis while he had interrupted follow-up. There was no decrease in the incidence of a second TCGT with time and the longest interval between tumours was 20 years. Intervals as long as 32 years between tumours were reported in one series (Schreiber *et al*, 1987). Consequently, patients with TGCT require life-long follow-up with periodic self-examination and ultrasonography, which is a sensitive and reliable detection method (Patel *et al*, 1990, 1993).

The treatment decisions for the second tumour were determined mainly according to the clinical and pathological characteristics of the second tumour and, sometimes, according to the treatment of the first tumour. Only two patients had a repeated retroperitoneal lymph-node dissection. In all, 11 patients were offered surveillance for low-risk stage I seminoma or NSGCT. One of them relapsed and was rendered disease free with chemotherapy.

Five patients treated after 2001 underwent conservative surgery. The adjuvant treatments were different and were according to pathological findings and at the physician's discretion. Testis-preserving surgery is more and more widely used for patients with BGCT (Heidenreich *et al*, 1995, 1997, 2000; Mearini *et al*, 1996; Fernandez Aparicio *et al*, 2000; Kirkali *et al*, 2001). A 20 Gy irradiation dose to the remaining testis has been reported to be a safe approach (Petersen *et al*, 2002). However, local relapses have also been reported following this treatment modality (Dieckmann *et al*, 1993, 1999, 2002). Neoadjuvant or adjuvant chemotherapy have been used to permit conservative treatment. (Oliver *et al*, 1996; Sawyer *et al*, 1999). Testicular relapses were observed in that series of patients, but had no impact on survival. Apparently, none of the conservative strategies are totally safe, but organ-sparing surgery in patients with BGCT offers endocrinological and psychological advantages. It should only be offered to patients who are aware and accept the risk of a subsequent local relapse and who realise the importance of compliance during follow-up.

Testicular intratubular germ-cell neoplasia is considered a precursor lesion for developing germ-cell tumours. It has been reported that at least 5% of patients with testicular germ-cell tumour have intratubular germ-cell neoplasia in the contralateral biopsy specimen when they undergo orchidectomy (Dieckmann *et al*, 1993, 1999, 2002; Daugaard *et al*, 1996; Herr and Scheifeld, 1997). Consequently, it has been suggested that patients with unilateral germ-cell tumours should undergo a systematic contralateral testicular biopsy at the time of orchidectomy to evaluate intratubular germ-cell neoplasia and identify patients who may develop a second germ-cell tumour (Dieckmann *et al*, 1993, 1999, 2002; Daugaard *et al*, 1996; Harland *et al*, 1998). However, the 1.3% incidence rate that we found for metachronous BGCT is significantly different compared with the reported incidence of intratubular germ-cell neoplasia if systematic contralateral biopsies are performed. This suggests that intratubular germ-cell neoplasia occurs more frequently than invasive germ-cell tumours. It has been reported that about 50% of patients with intratubular germ-cell neoplasia who do not receive radiation therapy to the testis nor systemic chemotherapy will develop an invasive germ-cell tumour at 5 years (Montinori, 2002). Since a contralateral biopsy has no adverse effect in itself, it could be recommended as a routine procedure. However, this procedure may lead to a false sense of safety since false-negative biopsy results have been reported (Dieckmann *et al*, 1993, 1999, 2002). The same surveillance would therefore have to be recommended for patients with negative biopsy results. Furthermore, there is no consensus about the management of patients with intratubular neoplasia. Whether all patients with germ-cell intratubular neoplasia should receive radiotherapy to the contralateral testis remains debatable. The extent of radiotherapy in this indication is also controversial, (Classen *et al*, 2000; Sedlmayer *et al*, 2001; Petersen *et al*, 2002). Delivering doses below 20 Gy is not considered safe and local relapses have been reported even at this dose (Dieckmann *et al*, 1993, 1999, 2002). Although the fertility of patients with intraepithelial germ-cell neoplasia is generally poor, it has been reported to be sufficient in some patients to father a child (Bokemeyer *et al*, 1993; Jacobsen *et al*, 2002), while radiotherapy to the contralateral testis results in definitive infertility. Radiotherapy may also affect Leydig cell function, even at the lowest doses (Von der Maase *et al*, 1986; Dieckman *et al*, 1993, 1999, 2002; Petersen *et al*, 2002).

Close clinical follow-up may therefore be recommended for patients with a unilateral germ-cell tumour and characteristics such as microlithiasis at ultrasonography (Berger and Brabrand, 1998) or testicular atrophy or impaired gonadal function (Harland *et al*, 1998), which lead to suspicion of contralateral intraepithelial neoplasia, since there is concern about the effect of radiotherapy. Chemotherapy has also been reported to lower the incidence of metachronous BGCT, with an estimated risk of recurrent intraepithelial germ-cell neoplasia of 21 and 42% at 5 and 10 years, respectively (Oliver *et al*, 1996). This finding associated with the long-term experience with single-agent carboplatin for clinical stage I seminoma (Steiner *et al*, 2002) may lead to recommendations for treatment with this cytotoxic agent rather than radiotherapy for patients presenting with seminoma below 30 years of age.
